# Noise-induced instability of uniform flow in single-file traffic systems

**DOI:** 10.1093/pnasnexus/pgag101

**Published:** 2026-04-03

**Authors:** Oscar Dufour, Alexandre Nicolas, David Rodney, Jakob Cordes, Andreas Schadschneider, Antoine Tordeux

**Affiliations:** Université Claude Bernard Lyon 1, CNRS, Institut Lumière Matière, UMR 5306, F-69100 Villeurbanne, France; Université Claude Bernard Lyon 1, CNRS, Institut Lumière Matière, UMR 5306, F-69100 Villeurbanne, France; Université Claude Bernard Lyon 1, CNRS, Institut Lumière Matière, UMR 5306, F-69100 Villeurbanne, France; Institut für Theoretische Physik, Universität zu Köln, 50937 Cologne, Germany; Institute of Advanced Simulation, Forschungszentrum Jülich GmbH, 52428 Juelich, Germany; Institut für Theoretische Physik, Universität zu Köln, 50937 Cologne, Germany; Institut für Physikdidaktik, Universität zu Köln, 50931 Cologne, Germany; Fakultät für Maschinenbau und Sicherheitstechnik, Institute of Mathematical Modelling, Analysis and Computational Mathematics, Bergische Universität Wuppertal, 42119 Wuppertal, Germany

**Keywords:** vehicular traffic, stochastic instabilities, statistical physics

## Abstract

Stop-and-go waves in vehicular traffic are commonly explained as a linear collective instability induced by, eg response delays. We explore an alternative mechanism that more faithfully mirrors oscillation formation in dense single-file traffic. The fluctuating drivers’ responses, modeled by noise, play a key role in this model; as noise is increased, the base (uniform) flow abruptly switches to stop-and-go dynamics despite its unconditional linear stability. The stop-and-go motion persists only as long as noise is present. We rationalize the instability mechanism in quantitative terms by breaking down the noise into oscillatory driving terms that can be studied separately. Finally, we hint at an analogy with Kapitza’s inverted pendulum.

Significance statement Phantom jams in dense car traffic are a problem of both fundamental and practical relevance. We contribute to the elucidation of their emergence by quantitatively rationalizing a noise-induced instability mechanism that faithfully reproduces empirical observations of oscillations in single-file traffic. This instability is stochastic in essence, nonperturbative, and nonlocal. Moreover, the conventional methods of thermodynamics are of little avail because the system is out-of-equilibrium, with nonreciprocal interactions. Instead, we introduce and build on an analogy with the stability switch in a (Kapitza) pendulum under vibrations, which may be relevant for a broader class of systems.

## Introduction

Analyzing the stability of many-body systems (N⩾3) is often a prickly issue, as illustrated by the longstanding debate over the stability of the solar system. Further complications arise when the system’s dynamics cannot be described by deterministic equations of motion (1). Vehicular traffic perfectly illustrates this difficulty, and we will use this illustration, even though our findings have bearing on a broader class of traffic-like systems. Although every driver is familiar with stop-and-go waves on highways (where traffic jams, sometimes called phantom jams, emerge for no apparent reason, forcing drivers to slow down and speed up again), the underlying physical origin is still a matter of debate, even for single-file traffic. The debate has practical implications for safety and congestion, recently reignited by the instabilities observed in platooning experiments with vehicles equipped with adaptive cruise control (ACC) systems (2–4).

A plethora of factors can destabilize single-file traffic flow, as vehicle control and environmental perception are subject to reaction times, delays (5), and inaccuracies in perception or response (6, 7). Several of these factors can be handled deterministically. In particular, finite response times and latency in vehicle control (5, 8–12) can be modeled by inertial ordinary differential equations (13) and delayed linear equations (5). Both theoretical (9, 13–18) and numerical studies (19, 20) have traditionally identified the foregoing factors as causes of (linear) instabilities that give rise to stop-and-go dynamics that ensue when the response becomes too slow (21), in line with experimental results on ACC-equipped vehicles (3, 4), notably for the fine-tuned nonlinear models (22–25).

However, for cars without ACC, these delay-induced instabilities arguably fail to capture prominent observations in naturalistic (26, 27) or purely experimental (26, 28–31 ) conditions. In experimental “ring” scenarios (30, 31), around 20 drivers followed each other in single file along a large loop. Above a critical density, stop-and-go waves emerged. However, rather than arising from the gradual amplification of an initial perturbation, they appeared after stochastic incubation periods ranging from one to several minutes (2, 30). Such irregular emergence of stop-and-go waves is incompatible with linear instability. Instead, it points to a metastable (and *not* linearly unstable) state (32) (33, Chapter 6.3-5). Moreover, linearly unstable systems that directly relate speed and spacing cannot account for the fact that speed oscillations are not strongly amplified along a platoon of cars following a leader driving at constant speed, but follow a concave curve (26, 29, 34). In contrast, concave growth can be almost quantitatively reproduced by car-following models featuring (deterministic) action points, ie in which a response is triggered only if the stimulus exceeds a finite threshold (34).

Alternatively, this concave growth of fluctuations is equally well reproduced by complementing the equation of motion with a stochastic term (26, 34), viz.


(1) $$dv_{n}(t)=F\left(\Delta x_{n}(t),v_{n}(t),v_{n+1}(t)\right)\;dt+\sigma \;dW_{n}(t).$$

The stochastic forcing $\sigma \;dW_{n}$ describes the combined effect of human error and of the many degrees of freedom left out of the deterministic response *F*, which here depends on the gap $\Delta x_{n}=x_{n+1}-x_{n}-\ell$ ($x_{n}$ is the position of the *n*th car, $x_{n+1}$ the position of the predecessor, and ℓ≥0 the car length) and the speeds $v_{n}={\dot{x}}_{n}$ and $v_{n+1}={\dot{x}}_{n+1}$. For systems exhibiting linear instability over some range of parameters, a pronounced effect of the noise is expected. Indeed, even in strictly physical systems (35), “noisy precursors” arise near dynamical instabilities in the form of eg sustained oscillations. These oscillations are also seen in *stable* linear car-following models, in which noise is added to account for human error or idiosyncrasies (7, 26). But they should not be mistaken for a genuine instability: *additive* noise cannot modify the stability of a linearized stochastic differential equation (1). Instead of additive noise, a *multiplicative* noise was employed in Ref. (36), which grows with the relative speeds. Crucially, in these models, the noise acts *perturbatively*, which makes analytical advances possible. By contrast, qualitative reports of a sudden nucleation of stop-and-go waves amid stationary flow, triggered by random fluctuations (37–40), remain theoretically opaque.

In this Letter, we shed quantitative light on this alternative destabilization mechanism. By injecting simple additive noise into a car-following model that is unconditionally linearly stable in the deterministic limit, we ascertain the idea that noise per se can induce inherently nonperturbative stop-and-go-waves. We elucidate the nonlinear instability mechanism by drawing on an analogy with the Kapitza pendulum. This finding calls into question the widespread tendency in the literature to focus primarily on the *local* properties of the deterministic response function *F* (34, 36).

## Results

Traffic problems have given rise to models galore. Insight into metastable states was gained in part using cellular automata and interacting particle systems (41–44), (33, Chapter 8.1). Instead, here we target continuous (in time and space) car-following models of the generic structure of Eq. 1, which can be either deterministic ($dW_{n}=0$) or stochastic. We have studied a number of models in this broad class, including the stochastic full velocity difference (SFVD) model (45–48), the inertial car-following model of Tomer et al. (24), and the stochastic intelligent driver (SID) model (34), detailed in the Materials and methods section. Our focus will be put here on the stochastic adaptive time gap (SATG) model, whose basic dynamics are given by


(2) $$dv_{n}=\frac{1}{T_{n}}\left(\lambda \left(\Delta x_{n}-Tv_{n}\right)+\Delta v_{n}\right)\;dt+\sigma \;dW_{n},$$

where T>0 denotes the desired time gap and λ>0 is a sensitivity parameter. The deterministic part of the model follows from the relaxation dynamics of the time-gap variable $T_{n}=\Delta x_{n}/v_{n}$, defined as the ratio between the gap and the speed, and governed by ${\dot{T}}_{n}=\lambda (T-T_{n})$. Similarly to classical linear optimal velocity (OV) and Helly-type models (19, 23, 49), the resulting ATG model (2), written in Newtonian form, involves two relaxation mechanisms: one adjusting the speed according to the gap, and another relaxing the speed difference with respect to the preceding vehicle. However, in the ATG model the relaxation time is itself given by the time-gap variable, which makes the dynamics nonlinear. Although this modeling feature may appear minor, it plays a crucial role in the stability of the flow under stochastic perturbations. To regularize the equation near collisions ($\Delta x_{n}\to 0$) and complete stops ($v_{n}\to 0$), we bound $T_{n}$ using a smooth maximum (respectively, minimum) function. Other regularizations are possible; they affect the way stop-and-go waves unfurl after they have emerged, but not the process of their formation. Details are given in Appendix A (SI). These models are used to simulate a single file of cars (no overtaking) on a periodic circuit mimicking the experimental settings of Refs. (30, 31) (see Materials and methods section for details).

Linear stability analysis predicts that the regime of uniform, time-independent flow is stable at low car densities (for vanishing noise *σ*) but gives way to an instability (resulting in stop-and-go waves) at higher density in almost all models, at least for some range of parameters (8, 34). The SATG model stands out in this respect, being unconditionally linearly stable around the uniform flow state (see Appendix B (SI) and Refs. (50, 51)).

**Figure 1:**
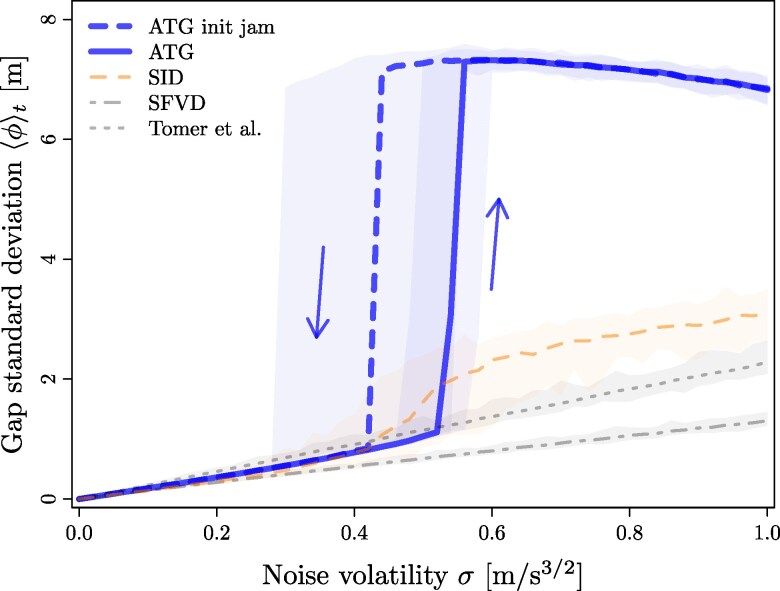
SD $\langle \phi \rangle_{t}$ (where $\langle \cdot \rangle_{t}$ denotes a time average) of the gaps in the stationary state as a function of the noise volatility *σ*, for N=22 cars in the experimental settings of (30) (see Materials and methods section for details). The overlays indicate the min/max range. The thick dashed line represents SATG simulations starting from a jammed initial condition, pointing to significant hysteresis.

**Figure 2:**
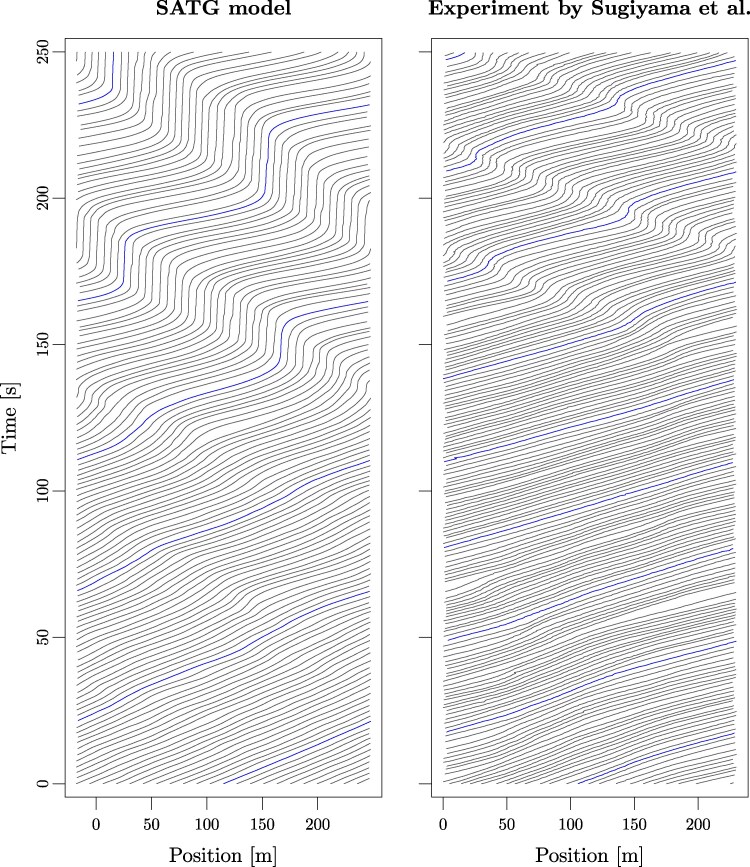
Trajectories, featuring stop-and-go waves for a selected simulation of the SATG model (10)–(11) with $\sigma =0.6\;m/s^{3/2}$ (left panel), and in the experiment of Sugiyama et al. (30) (right panel). A more realistic reproduction of the experiment using a fine-tuned ATG model is presented in Appendix A and Figure S1 of the Supplementary Information.

Direct numerical simulations of the models for N=22 cars confirm the expected stability for low noise *σ*. This is shown in Figure 1 using the disorder parameter $\phi (t)=\sqrt{\bar{\Delta x_{n}^{2}}(t)-{\bar{\Delta x_{n}}}^{2}(t)}$, where the overline denotes an average over the *N* cars. The gradual increase of the time average $\langle \phi \rangle_{t}$ with *σ* in the SFVD and Tomer models reflects the increasing fluctuations in intervehicular gaps induced by noise. In the SID model, disorder rises more markedly (but still fairly smoothly) around $\sigma \approx 0.5\;m/s^{3/2}$, mirroring the excitation of waves in the vicinity of a deterministic instability. In contrast, simulations of SATG reveal an unexpected, abrupt transition to traffic oscillations (high *ϕ* values) when *σ* crosses a threshold $\sigma^{⋆}$ ($\sigma^{⋆}$ decreases with increasing system size, at constant density, but rapidly converges to a finite threshold). For $\sigma >\sigma^{⋆}$, once developed, the stop-and-go wave is similar in shape to those found in experiments (see Figure 2 in the Materials and methods section; also see Figure S1 (SI) for fine-tuned model parameters) and in other models.

**Figure 3:**
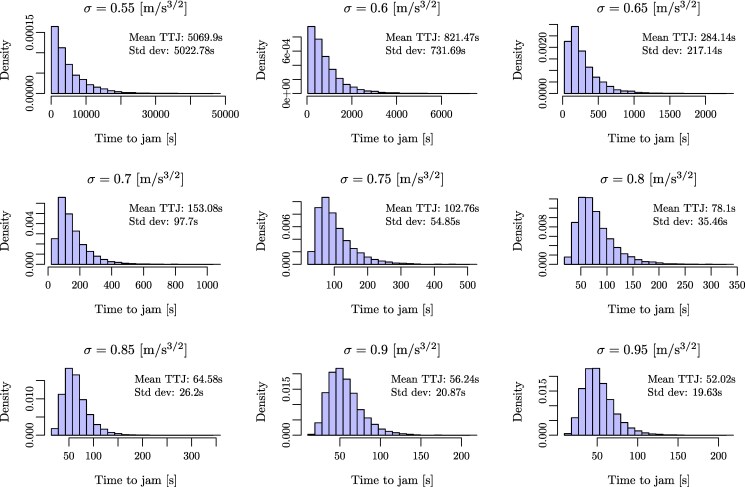
Histograms of the time for a stop-and-go wave to appear (TTJ) for a system with random initial conditions for noise volatilities ranging from 0.55 to $0.95\;m/s^{3/2}$. The more volatile the noise, the faster the waves appear.

The SATG route to instability also stands apart in that stop-and-go waves emerge erratically after a typically long but broadly varying transient time (Figure 3 in the Materials and methods section), unlike the waves originating from a linear instability. Such a transient is consistent with the empirical data of Ref. (30, 31), where stop-and-go waves do not occur systematically for a given car density. It points to a first-order transition toward traffic instability and to metastability of both the jammed and unjammed states, in accordance with the empirical conclusions of Ref. (32) and the numerical finding of a bimodal distribution of *ϕ* at the transition, in Figure S3 (SI). This also entails that the out-of-equilibrium SATG instability will not be tractable with common approaches such as continuation methods (52) or perturbative stochastic stability analysis (1), for want of a bifurcation in the deterministic limit.

As a corollary of metastability, hysteresis—a common observation in highway traffic (6, 53)—is observed when the system starts from the stop-and-go regime and the noise volatility (or the car density) is gradually reduced, as shown in Figure 1: stop-and-go waves subsist below the noise level required to destabilize the homogeneous flow. But they eventually subside and vanish before reaching σ=0. This implies that noise does not simply trigger the instability, as in a subcritical hydrodynamic instability but is required to sustain the stop-and-go dynamics, in the same way as temperature is needed to stabilize the gas phase past the boiling transition.

We hypothesize that the (finite) noise operates as an external driving force whose continuous actions push some system variables (in particular, the gaps $\Delta x_{n}$ here) beyond their stable range and ultimately stabilize stop-and-go dynamics. To make the idea more concrete, let us consider a realization of the noise $\left(\sigma dW_{n}\right)$ over a large time interval and split it into its Fourier modes. Separately, each mode $\left\{C_{n}\cos\;(\omega t+\varphi_{n})\right\}$, with car-dependent amplitudes $C_{n}>0$ and phases $\varphi_{n}$, exerts an oscillatory driving on the deterministic system, viz.,


(3) $${\dot{v}}_{n}(t)=F_{n}(t)+C_{n}\cos\;(\omega t+\varphi_{n}),$$

where $F_{n}(t)=\;:\;F(\Delta x_{n}(t),v_{n}(t),v_{n+1}(t))$. In Appendix D (SI), we show that, at the linear level, high-frequency modes have virtually no influence on the gaps (because of the finite response time), whereas low-frequency vibrations (ω→0) all have an equal impact on the mean-square gap fluctuations (proportional to $\sum_{n}C_{n}^{2}$); the crossover regime is narrow, extending over barely a decade with our reference parameters. Accordingly, it makes sense to try to reduce the effect of the noise to one suitably weighted, low-frequency oscillatory mode (of amplitude C>0), supplemented with a residual signal that will be handled as perturbative white noise $\hat{\sigma }\;d{\hat{W}}_{n}$, ie to approximate (2) with


(4) $$dv_{n}(t)=F_{n}(t)\;dt+C\cos\;(\omega t+\varphi_{n})\;dt+\hat{\sigma }\;d{\hat{W}}_{n}(t).$$

**Figure 4:**
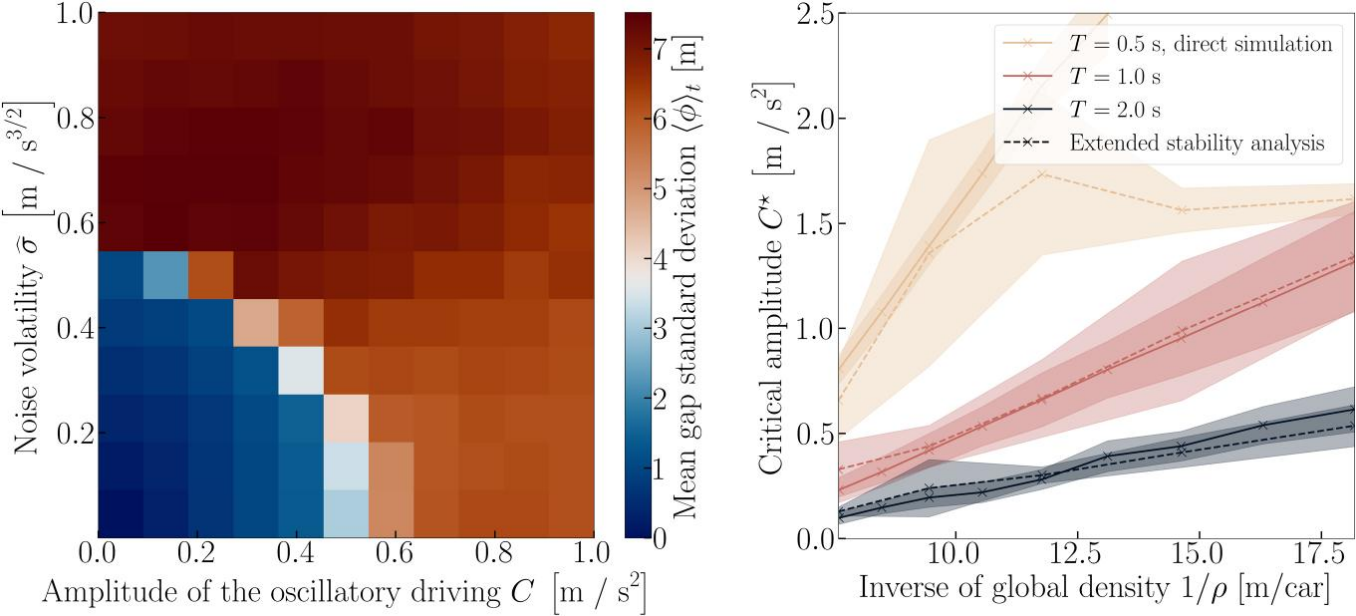
Left panel: Stability diagram in which the noise effect is heuristically split into a controlled oscillatory driving of amplitude *C* and a residual random noise $\hat{\sigma }$. Color overlay: SD of the gaps. Right panel: Variation of the stability threshold $C^{⋆}$ with the car density, computed with direct simulations of (4) at a driving frequency of 0.05⋅2πrad/s or derived from an extended stability analysis (see main text).

The numerical simulations presented in Figure 4, left panel, confirm that oscillatory driving facilitates the emergence of stop-and-go waves: the larger the driving amplitude *C*, the smaller the perturbative noise to trigger the instability. For strong enough driving $C⩾C^{⋆}$ (with $C^{⋆}=0.55\;m/s^{2}$ on Figure 4), the system becomes unstable under vanishing noise.

A theoretical prediction of the relation between $C^{⋆}$ and $\sigma^{⋆}$ is obtained in Appendix D (SI) by equating the mean-square gap fluctuations $\left\langle |\Delta x_{n}{|}^{2}\right\rangle$ expected for oscillatory driving and for white noise, after linearizing the equations. We arrive at $C^{⋆}=A(\lambda ,T)\sigma^{⋆}$, with a coefficient A(λ,T) of order $1\;s^{-1/2}$ that may depend on model parameters *λ* and *T*. In practice, the expression satisfactorily captures the insensitivity of A(λ,T) to car density and frequency, and its variations with model parameters, but only roughly approximates its numerical value.

With the oscillatory driving parallel in mind, the instability is now amenable to quantitative rationalization. At ω=0, the driving is equivalent to acceleration offsets $b_{n}=C\cos\;(\varphi_{n})$ applied to each car. It turns out that these heterogeneous offsets can make the system linearly unstable for a range of offsets $b_{n}$, and implicit analytical expressions can be derived for the growth rates, as we show in Appendix C (SI). Let $\nu (\{b_{n}\})$ be the largest nontrivial growth rate (ie the real part of the eigenvalue). If the oscillatory frequency *ω* was chosen small but nonzero, the driving can be treated as quasistationary. Under this approximation, at each time $t^{\prime }$, the peak perturbation grows exponentially as $\exp\;(\nu_{t^{\prime }}t)$, where $\nu_{t^{\prime }}=\nu (\{C\cos\;(\omega t^{\prime }+\varphi_{n})\})$. To go further, since the peak growth mode mostly spans the system size, we overlook the fact that it may change with time $t^{\prime }$, so that the effective growth rate over a cycle is


(5) $$\begin{array}{rl} \nu_{\mathrm{eff}}(C) & =\langle \nu_{t^{\prime }}\rangle_{t^{\prime }}\\ & ≃\langle \nu (\{C\;\cos\;(\theta_{n})\})\rangle_{\theta }, \end{array}$$

where the angular brackets denote averages over time $t^{\prime }$ or over uniformly distributed $\left\{\theta_{n}\right\}$. In the last approximation, we replaced the actual phases $\omega t^{\prime }+\varphi_{n}$ with random ones $\theta_{n}$ drawn from a uniform distribution over ]−π,π]. This yields an excellent agreement after averaging over the random phases $\varphi_{n}$, as observed numerically in Figure S4 (SI). The thresholds $C^{⋆}$ at which $\nu_{\mathrm{eff}}(C)$ becomes positive are determined by numerically computing the eigenvalues of Eq. 5. Remarkably, Figure 4, right panel, proves that the values of $C^{⋆}$ predicted by the present extended stability analysis accurately reproduce the instability thresholds measured in direct simulations of (4) under oscillatory driving, even at finite frequencies (deviations are observed for small *T* at low density). It follows that these calculations succeed in inferring the noise threshold $\sigma^{⋆}=C^{⋆}/A$, with $A=1.0\;s^{\;}\text{-}1/2$, at which the genuine SATG model (Eq. 2) undergoes a transition (Figure 1).

## Discussion

Taking a step back, we notice that the mechanism by which the instability unfolds in the SATG model is analogous to the switch of stable positions in a Kapitza pendulum. Indeed, while the bottom equilibrium of a rigid pendulum is unconditionally stable at rest, strong enough vertical vibrations may destabilize it and stabilize the “inverted” state (here amalgamated with the state of stop-and-go waves).

**Figure 5:**
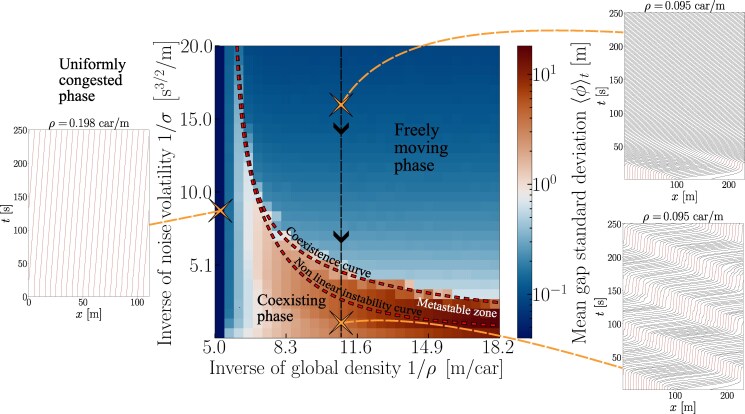
*Main panel:* “Phase” diagram of SATG traffic as a function of the inverse car density (ie the distance between the midpoints of cars) and the inverse noise volatility; the heat map represents the gap SD ⟨ϕ⟩. The side panels show kymographs of trajectories in different regions of parameter space.

Despite the singularity of this destabilizing mechanism, from a broader perspective, we find that the general picture of a first-order phase transition established for traffic instabilities (54, 55) originating from other processes, eg reaction delay (56), pinch and nucleation effects in the three-phase flow theory (57, 58), slow-to-start effects (41), or cellular automata simulated in continuous space (38), still holds. To clarify the picture, let us map the transition to traffic oscillations onto a liquid-gas transition (59) by assimilating the noise volatility *σ* to the temperature and the inverse headway (local density *ρ*) between cars to the density. In Figure 5, we plot the “phase” diagram of the disorder parameter *ϕ* as a function of *σ* and *ρ*. A line of discontinuous transitions is clearly observed at intermediate densities for all “temperatures” *σ above* a critical value, while the homogeneous state remains stable at both low (gas-like) and high (liquid-like) densities. Amusingly, in this liquid-gas analogy, the gas phase begins to “boil” as the temperature *σ* increases—a counterintuitive phenomenon reminiscent of a paradox found in simple crowd models (60).

Finally, we use the image of a liquid–gas transition to shed a different light on the hysteresis observed in Figure 1. When *σ* is varied at a fixed density, as considered in Figure 1, the system moves along a vertical line in Figure 5: it does not transit between two pure states, but rather from a pure phase into a coexistence region. Starting from the uniform regime, the system may avoid developing an instability until it hits the spinodal. In contrast, starting from the coexistence region, stop-and-go waves may persist up to a different neutral stability line, known as the binodal line. Interestingly, unlike in a liquid–gas mixture below the binodal line, there is no *static* coexistence between the jammed and freely flowing phases in a steady state; instead, the jam moves continuously. Similar transitions (61) into circulating states were recently identified in systems with nonreciprocal interactions (62); this is easily rationalized by considering that each car is tied by a spring to the predecessor, but not to its follower, so that the in and out currents cannot compensate at an interface.

In conclusion, we have shown that the SATG model can undergo an abrupt transition into a state characterized by stop-and-go waves similar to experiments (31). Interestingly, the model is unconditionally stable and the transition is not the result of a linear instability but rather the consequence of the continuous action of the noise, which (i) destabilizes the homogeneous state, (ii) stabilizes the heterogeneous state, and (iii) pushes the system between states. This mechanism is akin to the stability switch in a Kapitza pendulum. Thus, we surmise that the idea of substituting a simple oscillatory driving for stochastic forces with nonperturbative effects has broader applicability than that demonstrated here for SATG.

From an even broader perspective, the SATG stability diagram resembles that of a liquid–gas phase transition (albeit with an unsteady phase coexistence), as qualitatively discussed for previously evidenced instabilities, notably in deterministic car-following models with a reaction time (5) (where the reaction time plays the role of the temperature) or in the Nagel–Schreckenberg cellular automaton (63) and its continuous time-limit (37, 38), where at each time step cars may brake by a random amount (relative to the desired acceleration). In these scenarios as well as in SATG, the variables that elude a deterministic description (in particular, the “human error” (7), ie the inaccurate drivers’ perceptions or responses, their variability) play a central role. Macroscopic observations are insufficient to discriminate between the foregoing microscopic mechanisms, but virtual reality experiments could prove instrumental to disentangle these physical ingredients.

As a closing remark, note that the first-order transition picture need not be universal across all forms of traffic; for instance, pedestrian single-file traffic displays stop-and-go waves that are rather evanescent, considerably smaller than in highway traffic (64, 65), and not interspersed with freely flowing phases, unlike road traffic.

## Materials and methods

### Simulation settings

We simulate *N* cars on a single lane of length *L* with periodic boundary conditions. By default, we set N=22 and L=231m to mimic the experiments of (30, 31) at the onset of traffic oscillations. Cars are ordered from n=1 to n=N (n=1 is the predecessor of n=N because of the periodic boundaries); overtaking is precluded. At time *t*, car *n* is at position $x_{n}(t)$ and moves at speed $v_{n}(t)={\dot{x}}_{n}(t)$. The intervehicular spatial gap $\Delta x_{n}(t)$ and the speed difference $\Delta v_{n}(t)$ are given by


$$\left\{ \begin{array}{c} \Delta x_{n}(t)=x_{n+1}(t)-x_{n}(t)-\ell ,\;n\in \{1,\ldots ,N-1\},\\ \Delta x_{N}(t)=L+x_{1}(t)-x_{N}(t)-\ell , \end{array}\right.$$

where ℓ=5 m is the car length, and


$$\left\{ \begin{array}{c} \Delta v_{n}(t)=v_{n+1}(t)-v_{n}(t),\;n\in \{1,\ldots ,N-1\},\\ \Delta v_{N}(t)=v_{1}(t)-v_{N}(t), \end{array}\right.$$

respectively. The time dependencies will be dropped in the following.

### Car-following models

The following models have been implemented:

- the SFVD, as a representative of the class of (linear or near-linear) OV and full velocity difference models. It is given by Refs. (45–48)
  (6) $$dv_{n}=\left(\frac{V\left(\Delta x_{n}\right)-v_{n}}{T_{1}}+\frac{\Delta v_{n}}{T_{2}}\right)\;dt+\sigma \;dW_{n},$$
  where $T_{1}=2.5$ s and $T_{2}=2$ s are two relaxation times, and where $V\;:\;R\mapsto R_{+}$ is the OV function given by the sigmoid
  (7) $$V(s)=v_{0}\frac{\tanh\;(s/\ell_{0}-\kappa )+\tanh\;(\kappa )}{1+\tanh\;(\kappa )},$$
  where κ=0.5 and $\ell_{0}=20$ m are the shape and scale parameters, and where $v_{0}=20$ m/s is the desired speed (19, 23, 46).
- the stochastic (near-linear) model of Tomer et al. (24) given by
  (8) $$\begin{array}{rl} dv_{n} & =K\left(1-\frac{2v_{n}T+\ell }{\Delta x_{n}+\ell }\right)\;dt+\frac{Z^{2}\left(-\Delta v_{n}\right)}{2\Delta x_{n}}\;dt\\ & \;-2Z\left(v_{n}-v_{0}\right)\;dt+\sigma \;dW_{n}, \end{array}$$
  where Z(x)=(x+|x|)/2 is the positive part of *x*, $K=5\;m/s^{2}$ is a sensitivity parameter, and T=1 s is the desired time gap.
- the SID model (34), which reads
  (9) $$\begin{array}{c} dv_{n}=a\left(1-{\left(\frac{\;f\left(v_{n},\Delta v_{n}\right)}{\Delta x_{n}}\right)}^{2}-{\left(\frac{v_{n}}{v_{0}}\right)}^{4}\right)\;dt+\sigma \;dW_{n}\\ \text{with}\;f(v,\Delta v)=s_{0}+Tv-v\frac{\Delta v}{2\sqrt{ab}}, \end{array}$$
  where $a=b=2\;m/s^{2}$ are the desired acceleration and maximal deceleration parameter, $s_{0}=2$ m is a minimal gap, T=1 s is the desired time gap and where $v_{0}=20$ m/s is the desired speed.
- the SATG model, obtained by relaxing the time gap $T_{n}(t)=\Delta x_{n}/v_{n}$ as ${\dot{T}}_{n}(t)=\lambda (T-T_{n}(t))$, where $\lambda =0.2\;s^{-1}$ is a sensitivity parameter and T=1 s is the desired time gap (25). Using speed and gap variables, the model reads
  (10) $$dv_{n}=\frac{\lambda \left(\Delta x_{n}-Tv_{n}\right)+\Delta v_{n}}{T_{\varepsilon }\left(\Delta x_{n},v_{n}\right)}\;dt+\sigma \;dW_{n}.$$
  For practical purposes (namely, to avoid singularities when the cars are about to collide due to the noise or when their speed goes to zero), the time gap in the denominator is bounded between $T_{\min}=0.1$ s and $T_{\max}=4$ s using the smooth maximum (respectively, minimum)
  (11) $$T_{\varepsilon }(\Delta ,v)=f_{\varepsilon }\left(T_{\min},f_{-\varepsilon }\left(T_{\max},\frac{\Delta }{v}\right)\right),$$
  where $f_{\varepsilon }$ is the LogSumExp function $f_{\varepsilon }(a,b)=\varepsilon \log\;(e^{a/\varepsilon }+e^{b/\varepsilon })$. The function $f_{\varepsilon }(a,b)$ converges to the maximum of *a* and *b* as $\varepsilon \to 0^{+}$ and to the minimum as $\varepsilon \to 0^{-}$. In practice, ε is set to 0.01.

In all the above expressions, $\sigma \;dW_{n}$ represents the stochastic noise, where the $W_{n}(t)$ denote independent Wiener processes (see below for the actual implementation). The deterministic versions of the models are obtained by setting the volatility *σ* to 0.

### Numerical implementation

We used an implicit/explicit Euler–Maruyama numerical solver for the simulation with a time step δt=0.001 s. for the *n*th agent, n∈{1,…,N}, the numerical scheme reads


(12) $$\left\{ \begin{array}{c} x_{n}(t+\delta t)=x_{n}(t)+\delta tv_{n}(t+\delta t),\\ v_{n}(t+\delta t)=v_{n}(t)+\delta tF_{n}(t)+\sqrt{\delta t}g(v_{n}(t))\xi_{n}(t), \end{array}\right.$$

where $F_{n}(t)$ is used as a shorthand for the model-dependent deterministic response function $F(\Delta x_{n}(t),v_{n}(t),v_{n+1}(t))$, the $\xi_{i}(t)$’s (for i=1,…,N and t∈δtN) are independent normal random variables, and


(13) $$g(v)=\frac{\sigma }{1+\exp\;(-\alpha (v-v_{\sigma }))},\;\sigma \geq 0,\;\alpha =10^{3},$$

is designed to be close to zero when *v* gets smaller than $v_{\sigma }=0.1$ m/s to limit the collisions and equal to the volatility constant *σ* when $v\gg v_{\sigma }$.

### Distribution of times for the emergence of stop-and-go waves in SATG

In the ring experiment, the time for a stop-and-go wave to emerge is about 150 s in (30) and 320 s in (32). These times, called time-to-jam (TTJ), are also variable in the SATG model (10)–(11). Figure 3 shows the histograms of the TTJ for noise volatilities *σ* ranging from 0.55 to $0.95\;m/s^{3/2}$. $10^{4}$ simulations are repeated for each *σ*. The initial condition is uniform. We consider that a stop-and-go wave has emerged when the SD of gaps exceeds 6 m. The more volatile the noise, the faster the waves appear. The empirically observed variability seems to correspond to $\sigma \;(m/s^{3/2})\in [0.6,0.7]$.

### Computation of $\langle \phi \rangle_{t}$

In Figure 1, we run warm-up simulations for 5,000 s (to reach stationarity) before time-averaging the gap SD *ϕ* over the next 2,000 s. We repeat 100 independent Monte Carlo simulations for each value of the noise volatility ranging from 0 to 1 in steps of 0.02 and each of the four stochastic car-following models of Eqs. 6–10. In the figure, the curves are the median values of the Monte Carlo simulations, while the colored areas show the minimum/maximum range of variation.

## Supplementary Material

pgag101_Supplementary_Data

## Data Availability

The data presented in this study were generated through numerical simulations of the model fully specified in the manuscript, which provides all equations and parameters necessary for reproduction. In addition, an online simulation platform of the solver (12) for the setting of the experiment by Sugiyama et al. (30 ) and the stochastic car-following models of Eqs. 6–10 is available at https://vzu.uni-wuppertal.de/fileadmin/site/vzu/Experiment_by_Sugiyama_et_al._2007.html?speed=0.8.

## Preprints

This manuscript has been posted as a preprint at https://doi.org/10.48550/arXiv.2503.23594.

## References

[1] Gardiner C . Stochastic methods. Vol. 4. Springer, Berlin, 2009.

[2] Stern RE, et al 2018. Dissipation of stop-and-go waves via control of autonomous vehicles: field experiments. Transp Res Part C Emerg Technol. 89:205–221.

[3] Gunter G, et al 2020. Are commercially implemented adaptive cruise control systems string stable? IEEE Trans Intell Transp Syst. 22).6992–7003.

[4] Makridis M, Mattas K, Anesiadou A, Ciuffo B. 2021. OpenACC. An open database of car-following experiments to study the properties of commercial ACC systems. Transp Res Part C Emerg Technol. 125:103047.

[5] Nagatani T, Nakanishi K. 1998. Delay effect on phase transitions in traffic dynamics. Phys Rev E. 57:6415–6421.

[6] Yeo H, Skabardonis A. Understanding stop-and-go traffic in view of asymmetric traffic theory. In Transportation and traffic theory 2009: golden jubilee: papers selected for presentation at ISTTT18, a peer reviewed series since 1959. Springer, 2009. p. 99–115.

[7] Laval JA, Toth CS, Zhou Y. 2014. A parsimonious model for the formation of oscillations in car-following models. Transp Res B Methodol. 70:228–238.

[8] Orosz G, Wilson RE, Krauskopf B. 2004. Global bifurcation investigation of an optimal velocity traffic model with driver reaction time. Phys Rev E. 70:026207. 10.1103/PhysRevE.70.026207 15447565

[9] Orosz G, Wilson RE, Stépán G. 2010. Traffic jams: dynamics and control. Philos Trans R Soc A. 368:4455–4479. 10.1098/rsta.2010.0205 20819817

[10] Wilson RE, Ward JA. 2011. Car-following models: fifty years of linear stability analysis—a mathematical perspective. Transp Plan Technol. 34:3–18.

[11] Tordeux A, Roussignol M, Lassarre S. 2012. Linear stability analysis of first-order delayed car-following models on a ring. Phys Rev E. 86:036207. 10.1103/PhysRevE.86.036207 23030997

[12] Tordeux A, Costeseque G, Herty M, Seyfried A. 2018. From traffic and pedestrian follow-the-leader models with reaction time to first order convection-diffusion flow models. SIAM J Appl Math. 78:63–79.

[13] Komatsu TS, Sasa S. 1995. Kink soliton characterizing traffic congestion. Phys Rev E. 52:5574–5582. 10.1103/physreve.52.5574 9964055

[14] Reuschel A . 1950. Fahrzeugbewegungen in der Kolonne. Österr Ing Arch. 4:193–215.

[15] Pipes LA . 1953. An operational analysis of traffic dynamics. J Appl Phys. 24:274–281.

[16] Kometani E, Sasaki T. 1958. On the stability of traffic flow (report-I). J Oper Res Soc Jpn. 2:11–26.

[17] Chandler RE, Herman R, Montroll EW. 1958. Traffic dynamics: studies in car following. Oper Res. 6:165–184.

[18] Herman R, Montroll EW, Potts RB, Rothery RW. 1959. Traffic dynamics: analysis of stability in car following. Oper Res. 7:86–106.

[19] Bando M, Hasebe K, Nakayama A, Shibata A, Sugiyama Y. 1995. Dynamical model of traffic congestion and numerical simulation. Phys Rev E. 51:1035–1042. 10.1103/physreve.51.1035 9962746

[20] Bando M, Hasebe K, Nakanishi K, Nakayama A. 1998. Analysis of optimal velocity model with explicit delay. Phys Rev E. 58:5429.

[21] Makridis M, Mattas K, Ciuffo B. 2019. Response time and time headway of an adaptive cruise control. An empirical characterization and potential impacts on road capacity. IEEE Trans Intell Transp Syst. 21:1677–1686.

[22] Treiber M, Hennecke A, Helbing D. 2000. Congested traffic states in empirical observations and microscopic simulations. Phys Rev E. 62:1805–1824. 10.1103/physreve.62.1805 11088643

[23] Jiang R, Zuojin Zhu QW. 2001. Full velocity difference model for a car-following theory. Phys Rev E. 64:017101. 10.1103/PhysRevE.64.017101 11461442

[24] Tomer E, Safonov L, Havlin S. 2000. Presence of many stable nonhomogeneous states in an inertial car-following model. Phys Rev Lett. 84:382. 11015916 10.1103/PhysRevLett.84.382

[25] Tordeux A, Lassarre S, Roussignol M. 2010. An adaptive time gap car-following model. Transp Res B Methodol. 44:1115–1131.

[26] Jiang R, et al 2018. Experimental and empirical investigations of traffic flow instability. Transp Res Part C Emerg Technol. 94:83–98.

[27] Tian J, Jiang R, Jia B, Gao Z, Ma S. 2016. Empirical analysis and simulation of the concave growth pattern of traffic oscillations. Transp Res B Methodol. 93:338–354.

[28] Jiang R, et al 2015. On some experimental features of car-following behavior and how to model them. Transp Res B Methodol. 80:338–354.

[29] Tian J, et al 2019. On the role of speed adaptation and spacing indifference in traffic instability: evidence from car-following experiments and its stochastic model. Transp Res B Methodol. 129:334–350.

[30] Sugiyama Y, et al 2008. Traffic jams without bottlenecks—experimental evidence for the physical mechanism of the formation of a jam. New J Phys. 10:033001.

[31] Tadaki S-i, et al 2013. Phase transition in traffic jam experiment on a circuit. New J Phys. 15:103034.

[32] Nakayama A, et al 2009. Metastability in the formation of an experimental traffic jam. New J Phys. 11:083025.

[33] Schadschneider A, Chowdhury D, Nishinari K. Stochastic transport in complex systems. From molecules to vehicles. Elsevier, 2010.

[34] Treiber M, Kesting A. 2017. The intelligent driver model with stochasticity-new insights into traffic flow oscillations. Transp Res Proc. 23:174–187.

[35] Wiesenfeld K . 1985. Noisy precursors of nonlinear instabilities. J Stat Phys. 38:1071–1097.

[36] Ngoduy D . 2021. Noise-induced instability of a class of stochastic higher order continuum traffic models. Transp Res B Methodol. 150:260–278.

[37] Krauß S, Wagner P, Gawron C. 1996. Continuous limit of the Nagel-Schreckenberg model. Phys Rev E. 54:3707–3712. 10.1103/physreve.54.3707 9965521

[38] Jost D, Nagel K. Probabilistic traffic flow breakdown in stochastic car following models. In Hoogendoorn S.P., Luding S., Bovy P.H.L., Schreckenberg M., Wolf D.E., editors. Traffic and granular flow “03”. Springer, 2005. p. 88–103.

[39] Kerner BS . Understanding real traffic—paradigm shift in transportation science. Springer, 2021.

[40] Treiber M, Kesting A. Traffic flow dynamics. Springer Nature Switzerland, Cham, Switzerland, 2013.

[41] Barlovic R, Santen L, Schadschneider A, Schreckenberg M. 1998. Metastable states in cellular automata for traffic flow. Eur Phys J B. 5:793–800.

[42] Ke-Ping L, Zi-You G. 2004. Noise-induced phase transition in traffic flow*. Commun Theor Phys. 42:369–372.

[43] Kaupužs J, Mahnke R, Harris RJ. 2005. Zero-range model of traffic flow. Phys Rev E. 72:056125. 10.1103/PhysRevE.72.056125 16383706

[44] Huang Y-X, Guo N, Jiang R, Hu M-B. 2018. Instability in car-following behavior: new Nagel–Schreckenberg type cellular automata model. J Stat Mech. 2018:083401.

[45] Wagner P . 2011. A time-discrete harmonic oscillator model of human car-following. Eur Phys J B. 84:713–718.

[46] Treiber M, Helbing D. 2009. Hamilton-like statistics in onedimensional driven dissipative many-particle systems. Eur Phys J B. 68:607–618.

[47] Wang Y, Li X, Tian J, Jiang R. 2020. Stability analysis of stochastic linear car-following models. Transp Sci. 54:274–297.

[48] Friesen M, Gottschalk H, Rüdiger B, Tordeux A. 2021. Spontaneous wave formation in stochastic self-driven particle systems. SIAM J Appl Math. 81:853–870.

[49] Helly W Simulation of bottlenecks in single-lane traffic flow. In R. C. Herman, editor. Proceedings of the Symposium on Theory of Traffic Flow, Research Laboratories, General Motors. Elsevier, New York, NY, USA, 1959. p. 207–238.

[50] Khound P, Will P, Tordeux A, Gronwald F. 2023. Extending the adaptive time gap car-following model to enhance local and string stability for adaptive cruise control systems. J Intell Transp Syst. 27:36–56.

[51] Ehrhardt M, Tordeux A. 2024. Stability of heterogeneous linear and nonlinear car-following models. Franklin Open. 9:100181.

[52] Orosz G, Wilson RE, Krauskopf B. 2004. Global bifurcation investigation of an optimal velocity traffic model with driver reaction time. Phys Rev E. 70:026207. 10.1103/PhysRevE.70.026207 15447565

[53] Chen D, Laval JA, Ahn S, Zheng Z. 2012. Microscopic traffic hysteresis in traffic oscillations: a behavioral perspective. Transp Res B Methodol. 46:1440–1453.

[54] Kerner B, Rehborn H. 1997. Experimental properties of phase transitions in traffic flow. Phys Rev Lett. 79:4030–4033.

[55] Nagatani T . 2002. The physics of traffic jams. Rep Prog Phys. 65:1331–1386.

[56] Nagatani T . 1998. Thermodynamic theory for the jamming transition in traffic flow. Phys Rev E. 58:4271–4276.

[57] Kerner BS . The physics of traffic. Springer, 2004.

[58] Kerner BS . 1998. Experimental features of self-organization in traffic flow. Phys Rev Lett. 81:3797–3800.

[59] Nagel K, Wagner P, Woesler R. 2003. Still flowing: approaches to traffic flow and traffic jam modeling. Oper Res. 51:681–710.

[60] Helbing D, Farkas IJ, Vicsek T. 2000. Freezing by heating in a driven mesoscopic system. Phys Rev Lett. 84:1240–1243. 11017488 10.1103/PhysRevLett.84.1240

[61] Barkley D . 2016. Theoretical perspective on the route to turbulence in a pipe. J Fluid Mech. 803:P1.

[62] Fruchart M, Hanai R, Littlewood PB, Vitelli V. 2021. Non-reciprocal phase transitions. Nature. 592:363–369. 33854249 10.1038/s41586-021-03375-9

[63] Ke-Ping L, Zi-You G. 2004. Noise-induced phase transition in traffic flow. Commun Theor Phys. 42:369–372.

[64] Tordeux A, Schadschneider A. 2016. White and relaxed noises in optimal velocity models for pedestrian flow with stop-and-go waves. J Phys A Math Theor. 49:185101.

[65] Eilhardt C, Schadschneider A. Stochastic headway dependent velocity model and phase separation in pedestrian dynamics. In Chraibi M., Boltes M., Schadschneider A, Seyfried A., editors. Traffic and granular flow. Springer, 2015. 382.

