# Supplementary material for: Noise-induced instability of uniform flow in single-file traffic systems
Source: PNAS Nexus. 2026 Apr 3;5(4):pgag101. doi: 10.1093/pnasnexus/pgag101 (PMC13098188; doi:10.1093/pnasnexus/pgag101)
Supplement: pgag101_Supplementary_Data [file pgag101_supplementary_data.pdf]

Noise-induced instability of uniform flow in single-file traffic systems

Oscar Dufour, Alexandre Nicolas, and David Rodney
Université Claude Bernard Lyon 1, CNRS, Institut Lumière Matière, UMR 5306, F-69100, Villeurbanne, France

Jakob Cordes* and Andreas Schadschneider‡
Institut für Theoretische Physik, Universität zu Köln, Köln, Deutschland
**Also at Institute of Advanced Simulation, Forschungszentrum Jülich GmbH, Jülich, Deutschland*
‡*Also at Institut für Physikdidaktik, Universität zu Köln, Köln, Deutschland*

Antoine Tordeux
Fakultät für Maschinenbau und Sicherheitstechnik,
Bergische Universität Wuppertal, Wuppertal, Deutschland

(Dated: February 15, 2026)

A. NUMERICAL SIMULATIONS OF THE SATG MODEL

Fine-tuning SATG model to reproduce the stop-and-go waves observed in Sugiyama's experiment Figure S1 shows the simulated trajectories with a fine-tuned SATG model (Eq. 9-10) with $\sigma = 1$ m/s$^{3/2}$ that better reproduces the stop-and-go dynamics observed in Sugiyama's experiment [1]. The time gap is set to $T = 0.7$ s in order to match the task given to the drivers during the experiment (driving at a speed of 30 km/h, which corresponds to a time gap of 0.7 seconds, given the density). In addition, the maximum time gap $T_{\max} = 2$ s was decreased to reduce the duration of the stopping phase.

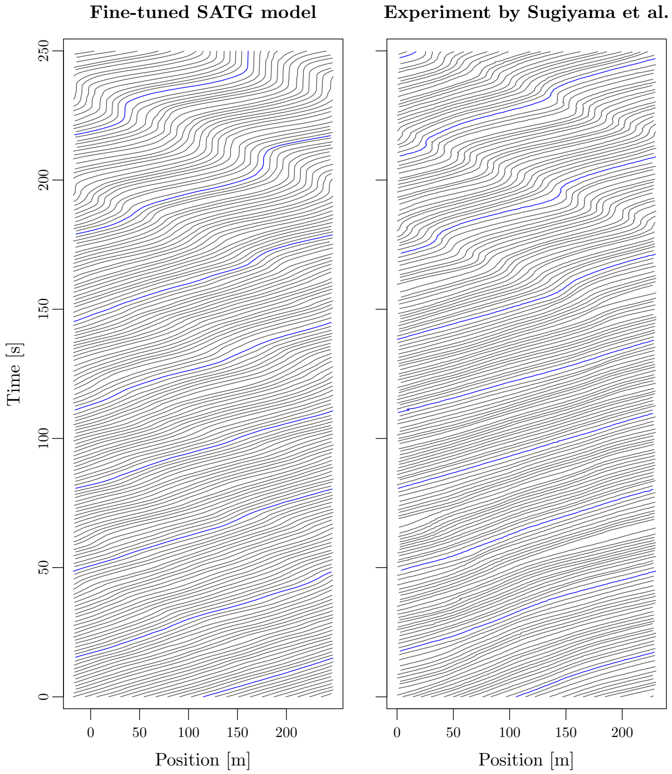

FIG. S1. Trajectories, featuring stop-and-go waves with a selected simulation of the fine-tuned SATG model with $T = 0.7$ s, $T_{\max} = 2$ s and $\sigma = 1$ m/s$^{3/2}$ (left panel) and in the experiment of Sugiyama et al. [1] (right panel).

Smoothed versus unsmoothed SATG models. The Stochastic Adaptive Time Gap (SATG) model, obtained by relaxing the time gap $T_n(t) = \Delta x_n / v_n$ to a desired time gap parameter T, reads

$$\mathrm{d}v_n = \frac{\lambda\left(\Delta x_n - T v_n\right) + \Delta v_n}{T_\varepsilon\left(\Delta x_n, v_n\right)}\mathrm{d}t + \sigma \mathrm{d}W_n, \tag{S1}$$

For practical purposes (namely, to avoid singularities when the cars are about to collide due to noise or when their speed goes to zero), we chose to regularize the model by bounding the time gap in the denominator between $T_{\min} = 0.1$ s and $T_{\max} = 4$ s using the smooth maximum (respectively, minimum)

$$T_\varepsilon(\Delta, v) = f_\varepsilon\left(T_{\min}, f_{-\varepsilon}\left(T_{\max}, \frac{\Delta}{v}\right)\right), \tag{S2}$$

where f_ε is the LogSumExp function $f_\varepsilon(a, b) = \varepsilon \log\left(e^{a/\varepsilon} + e^{b/\varepsilon}\right)$. The function $f_\varepsilon(a, b)$ converges to the maximum of a and b as $\varepsilon \to 0^+$ and to the minimum as $\varepsilon \to 0^-$. In practice, ε is set to 0.01.

This regularization influences how stop-and-go waves propagate once they have formed, but not the process of their formation. In Figure S2, we present two sets of trajectories, obtained with the same random seed, obtained with the smoothed SATG model of Eq. (S1)-(S2) (left panel) and the native (non-smoothed) SATG model, given by

$$\mathrm{d}v_n = \frac{\lambda\left(\Delta x_n - T v_n\right) + \Delta v_n}{\Delta x_n / v_n}\mathrm{d}t + \sigma \mathrm{d}W_n, \tag{S3}$$

(right panel). We observe that both feature stop-and-go waves, which start to emerge at the very same time. This confirms that the regularization of SATG does not affect the *emergence* of the instability. However, as expected, after the instability emerges, the native (non-smoothed) SATG displays erratic behavior, consistently with the presence of a singularity in the equations when the gap between vehicles vanishes.

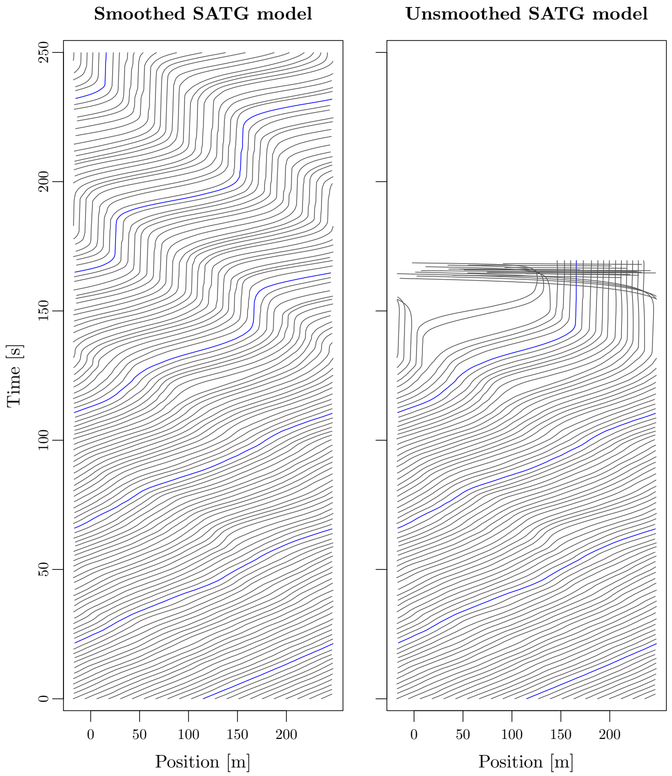

FIG. S2. Kymographs of trajectories simulated with (left panel) the smoothed SATG model of Eq. (S2) and (right panel) the non-smoothed SATG model of Eq. (S3), for parameters giving rise to featuring stop-and-go waves, using the same random seed. Trajectories coincide until the wave emerges and the gap of one the vehicle gets close to zero: regularization influences how stop-and-go waves unfurl once they have formed, but not the process of their formation.

Bimodality at the transition Figure S3 presents the distribution of the standard deviation of the gap at the critical volatility $\sigma = 0.56$ m/s$^{3/2}$ where the system is metastable (see Fig. 4). The system oscillates between a homogeneous state and stop-and-go dynamics at this noise level and the gap standard deviation has a bimodal distribution.

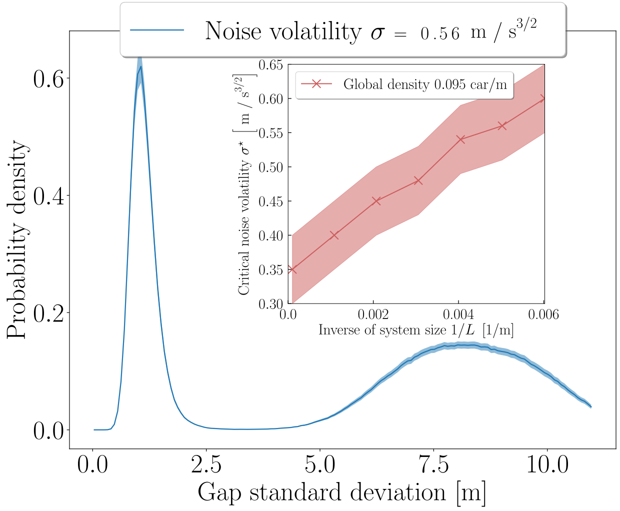

FIG. S3. Probability density function of the gap standard deviation ϕ over time and over independent simulations of the single-file periodic system with $N = 22$ cars close to the transition $\sigma = \sigma^\star$. *Inset:* Variation of the transition threshold $\sigma^\star$ with the (inverse) system size.

B. LINEAR STABILITY ANALYSIS OF THE BIASED DETERMINISTIC MODEL

This Appendix is dedicated to the linear stability analysis of the deterministic ATG model, either around the uniform stationary flow or around a heterogeneous state.

To explore the system's dynamics in the presence of heterogeneities, we bias the accelerations of the ATG model with time-independent offset terms b_n ($n \in \{1, \dots, N\}$) instead of the stochastic forcing, as follows:

$$\dot{v}_n(t) = F\big(v_n(t), \Delta x_n(t), \Delta v_n(t)\big) + b_n, \tag{S4}$$

where $v_n(t)$ is the speed, $\Delta x_n(t)$ is the gap, $\Delta v_n(t)$ is the speed difference to the predecessor and where

$$F(v, \Delta x, \Delta v) = \lambda v \left(1 - \frac{Tv}{\Delta x}\right) + \frac{v \Delta v}{\Delta x}, \tag{S5}$$

with $\lambda > 0$ the sensitivity parameter, T the desired time gap parameter, and b_n a constant and vehicle specific bias in the acceleration.

Equilibrium solution We consider $N \geq 2$ vehicle of length $\ell \geq 0$ on a ring of length $L > N\ell$. The equilibrium gap for the homogeneous system where $b_n = 0$ for all $b \in \{1, \dots, N\}$ is given by

$$g_\mathrm{e} = L/N - \ell. \tag{S6}$$

In the presence of acceleration biases, the equilibrium solution for which $\dot{v}_n = 0$ for all $n \in \{1, \dots, N\}$ is not uniform in space. This is the $\big(v_\mathrm{e}, (g_n^\mathrm{e})_{n=1}^N\big)$ configuration satisfying

$$\begin{cases} \displaystyle\sum_{n=1}^{N} g_n^\mathrm{e} = L - N\ell = N g_\mathrm{e}, \\ F(g_n^\mathrm{e}, v_\mathrm{e}, 0) + b_n = 0, \qquad n \in \{1, \dots, N\}. \end{cases} \tag{S7}$$

We can deduce from the second part that

$$g_n^{\mathrm{e}} = \frac{\lambda T v_{\mathrm{e}}^2}{b_n + \lambda v_{\mathrm{e}}}, \qquad \forall n \in \{1, \dots, N\}, \tag{S8}$$

while, using the conservation of spacing $\sum_n g_n^{\mathrm{e}} = N g_{\mathrm{e}}$, the equilibrium speed becomes the solution of

$$\sum_{n=1}^{N} \frac{\lambda T v_{\mathrm{e}}^2}{b_n + \lambda v_{\mathrm{e}}} = L - N\ell. \tag{S9}$$

Note that the equilibrium gaps (S8) are positive for all the vehicles if

$$v_{\mathrm{e}} > -\frac{1}{\lambda} \min_n b_n. \tag{S10}$$

In addition, we recover the equilibrium solution of the homogeneous ATG model

$$v_{\mathrm{e}} = \frac{g_{\mathrm{e}}}{T} \qquad \text{and} \qquad g_n^{\mathrm{e}} = g_{\mathrm{e}} \ \text{ for all } \ n \in \{1, \dots, N\}, \tag{S11}$$

if the biases are zero, i.e., $b_n = 0$ for all $n \in \{1, \dots, N\}$. Furthermore, in the case where the bias $b_n = b$ is identical for all vehicles $n \in \{1, \dots, N\}$, we have

$$\lambda T v_{\mathrm{e}}^2 - (b + \lambda v_{\mathrm{e}}) g_{\mathrm{e}} = 0, \tag{S12}$$

and we can deduce that

$$v_{\mathrm{e}} = \frac{g_{\mathrm{e}}\lambda + \sqrt{(g_{\mathrm{e}}\lambda)^2 + 4\lambda T b g_{\mathrm{e}}}}{2\lambda T} = \frac{g_{\mathrm{e}}}{2T}\left(1 + \sqrt{1 + \frac{4Tb}{\lambda g_{\mathrm{e}}}}\right) \tag{S13}$$

The equilibrium speed exists if $1 + \frac{4Tb}{\lambda g_{\mathrm{e}}} \geq 0$ and we obtain the condition

$$b \geq -\frac{\lambda g_{\mathrm{e}}}{4T}. \tag{S14}$$

Note that (S14) implies the preliminary condition $b > -\lambda v_{\mathrm{e}}$ (see (S10)).

Linearization of the system The partial derivatives of the model (S5) at equilibrium are given by

$$f_n^{\Delta x} = \frac{\partial F}{\partial g}(g_n^{\mathrm{e}}, v_{\mathrm{e}}, 0) = \frac{\lambda T v_{\mathrm{e}}^2}{(g_n^{\mathrm{e}})^2}, \qquad f_n^{v} = \frac{\partial F}{\partial v}(g_n^{\mathrm{e}}, v_{\mathrm{e}}, 0) = \lambda\left(1 - \frac{2T v_{\mathrm{e}}}{g_n^{\mathrm{e}}}\right), \quad \text{and} \quad f_n^{\Delta v} = \frac{\partial F}{\partial \Delta v}(g_n^{\mathrm{e}}, v_{\mathrm{e}}, 0) = \frac{v_{\mathrm{e}}}{g_n^{\mathrm{e}}} \tag{S15}$$

The characteristic equation of the linearised system is

$$\prod_{n=1}^{N} \left[z^2 - z(f_n^{v} - f_n^{\Delta v}) + f_n^{\Delta x}\right] - \prod_{n=1}^{N} \left[z f_n^{\Delta v} + f_n^{\Delta x}\right] = 0, \qquad \theta \in [0, 2\pi]. \tag{S16}$$

A general sufficient linear stability condition of a heterogeneous model for which all roots of (S16) have strictly negative real parts except one equal to zero (due to periodic boundary conditions) is [2, Eq. (5)]

$$\sum_{n=1}^{N} \left[\frac{1}{2}\left(\frac{f_n^{v}}{f_n^{\Delta x}}\right)^2 - \frac{f_n^{v} f_n^{\Delta v}}{f_n^{\Delta x} f_n^{\Delta x}} - \frac{1}{f_n^{\Delta x}}\right] \geq 0. \tag{S17}$$

We have

$$\frac{f_n^{v}}{f_n^{\Delta x}} = \frac{\lambda\left(1 - \frac{2T v_{\mathrm{e}}}{g_n^{\mathrm{e}}}\right)}{\frac{\lambda T v_{\mathrm{e}}^2}{(g_n^{\mathrm{e}})^2}} = \frac{g_n^{\mathrm{e}}(g_n^{\mathrm{e}} - 2T v_{\mathrm{e}})}{T v_{\mathrm{e}}^2}, \tag{S18}$$

while

$$\frac{f_n^v f_n^{\Delta v}}{f_n^{\Delta x} f_n^{\Delta x}} = \frac{\lambda \left(1 - \frac{2Tv_\mathrm{e}}{g_n^\mathrm{e}}\right) \frac{v_\mathrm{e}}{g_n^\mathrm{e}}}{\frac{\lambda^2 T^2 v_\mathrm{e}^4}{(g_n^\mathrm{e})^4}} = \frac{(g_n^\mathrm{e})^2 (g_n^\mathrm{e} - 2Tv_\mathrm{e})}{\lambda T^2 v_\mathrm{e}^3}. \tag{S19}$$

The sufficient linear stability condition (S17) is then given by

$$\sum_{n=1}^N \left[\frac{1}{2} \left(\frac{g_n^\mathrm{e}(g_n^\mathrm{e} - 2Tv_\mathrm{e})}{Tv_\mathrm{e}^2} \right)^2 - \frac{(g_n^\mathrm{e})^2 (g_n^\mathrm{e} - 2Tv_\mathrm{e})}{\lambda T^2 v_\mathrm{e}^3} - \frac{(g_n^\mathrm{e})^2}{\lambda T v_\mathrm{e}^2} \right] \geq 0, \tag{S20}$$

or again

$$\sum_{n=1}^N (g_n^\mathrm{e})^2 \left[\frac{\lambda (g_n^\mathrm{e} - 2Tv_\mathrm{e})^2}{2Tv_\mathrm{e}^2} - \frac{g_n^\mathrm{e} - 2Tv_\mathrm{e}}{Tv_\mathrm{e}} - 1 \right] = \sum_{n=1}^N (g_n^\mathrm{e})^2 \left[\frac{\lambda (g_n^\mathrm{e} - 2Tv_\mathrm{e})^2}{2Tv_\mathrm{e}^2} - \frac{g_n^\mathrm{e} - Tv_\mathrm{e}}{Tv_\mathrm{e}} \right] \geq 0. \tag{S21}$$

Then, using $g_n^\mathrm{e} = \frac{\lambda T v_\mathrm{e}^2}{b_n + \lambda v_\mathrm{e}}$ and remarking that $g_n^\mathrm{e} - 2Tv_\mathrm{e} = -Tv_\mathrm{e} \frac{2b_n + \lambda v_\mathrm{e}}{b_n + \lambda v_\mathrm{e}}$ while $g_n^\mathrm{e} - Tv_\mathrm{e} = -Tv_\mathrm{e} \frac{b_n}{b_n + \lambda v_\mathrm{e}}$, we obtain

$$\sum_{n=1}^N \left(\frac{\lambda T v_\mathrm{e}^2}{b_n + \lambda v_\mathrm{e}} \right)^2 \left[\frac{\left(Tv_\mathrm{e} \frac{2b_n + \lambda v_\mathrm{e}}{b_n + \lambda v_\mathrm{e}} \right)^2}{2Tv_\mathrm{e}^2} + \frac{Tv_\mathrm{e} \frac{b_n}{b_n + \lambda v_\mathrm{e}}}{Tv_\mathrm{e}} \right] \geq 0. \tag{S22}$$

After simplifications (we have $\lambda, T, v_\mathrm{e} > 0$), it follows

$$\sum_{n=1}^N \frac{1}{(b_n + \lambda v_\mathrm{e})^2} \left[\frac{\lambda T}{2} \left(\frac{2b_n + \lambda v_\mathrm{e}}{b_n + \lambda v_\mathrm{e}} \right)^2 + \frac{b_n}{b_n + \lambda v_\mathrm{e}} \right] \geq 0, \tag{S23}$$

or again

$$\sum_{n=1}^N \frac{1}{(b_n + \lambda v_\mathrm{e})^4} \left[\frac{\lambda T}{2} (2b_n + \lambda v_\mathrm{e})^2 + b_n (b_n + \lambda v_\mathrm{e}) \right] \geq 0. \tag{S24}$$

We have

$$\begin{aligned} \frac{\lambda T}{2} (2b_n + \lambda v_\mathrm{e})^2 + b_n (b_n + \lambda v_\mathrm{e}) &= 2\lambda T b_n^2 + \frac{1}{2} \lambda^3 T v_\mathrm{e}^2 + 2\lambda^2 T b_n v_\mathrm{e} + b_n^2 + b_n \lambda v_\mathrm{e} \\ &= b_n^2 (2\lambda T + 1) + b_n (2\lambda T + 1) \lambda v_\mathrm{e} + \frac{1}{2} \lambda^3 T v_\mathrm{e}^2 \\ &= b_n (2\lambda T + 1)(b_n + \lambda v_\mathrm{e}) + \frac{1}{2} \lambda^3 T v_\mathrm{e}^2, \end{aligned} \tag{S25}$$

and the linear stability condition can be written

$$\sum_{n=1}^N \frac{b_n (2\lambda T + 1)}{(b_n + \lambda v_\mathrm{e})^3} + \frac{\lambda^3 T v_\mathrm{e}^2}{2(b_n + \lambda v_\mathrm{e})^4} \geq 0. \tag{S26}$$

Since $\lambda, T > 0$, the model is linearly stable if $b_n = 0$ for all $b_n \in \{1, \dots, N\}$. Indeed the homogeneous ATG model is unconditionally linearly stable [3]. In addition, when all the biases are identical, i.e., $b_n = b > -\lambda g_\mathrm{e}/(4T)$ for all $b_n \in \{1, \dots, N\}$, dividing by $\lambda T v_\mathrm{e}^2 > 0$ the last line of (S25) and using (S13), we obtain the linear stability condition of the biased ATG model

$$\frac{b(2\lambda T + 1)}{g_\mathrm{e}} + \frac{\lambda^2}{2} \geq 0. \tag{S27}$$

This is

$$b \geq \frac{-\lambda^2 g_\mathrm{e}}{4\lambda T + 2}. \tag{S28}$$

The bias has to be negative and sufficiently low, especially for high λ or g_e (i.e., low density), to destabilise the system.

C. EXTENDED STABILITY ANALYSIS OF THE PERIODICALLY DRIVEN SYSTEM

This Section details the extended stability analysis of the car-following system in which the noise term is substituted by an externally applied deterministic oscillatory driving, with vanishing residual noise:

$$\dot{v}_n(t) = F\big(\Delta x_n(t), v_n(t), v_{n+1}(t)\big) + C\,\cos(\omega t + \varphi_n). \tag{S29}$$

We assume that the driving frequency is low enough so that a pseudo-stationary approximation can be performed, i.e., at each time t the system follows the biased deterministic equation of Eq. (S4) with $b_n = \cos(\omega t + \varphi_n)$. Perturbations around the base flow grow at rates ν given by the real parts of the eigenvalues z in Eq. (S16). Unfortunately, finding the roots z of this equation is far from straightforward analytically. The equation is thus solved numerically, first by deducing the equilibrium speed from Eq. (S9) and the associated gaps, and then finding the nontrivial complex roots $z \neq 0$ (the eigenvalue 0 is not relevant physically) of Eq. (S16) using a Newton-Raphson method with multiple starting points located on a regular lattice in complex space; we check that no eigenvalue has been missed by quadrupling the number of starting points. Numerically, identifying that z is a root of Eq. (S16) can be challenging for small λ or large T because a quasi-continuum of z values yield vanishingly small, but nonzero products. Accordingly, the validity of candidate roots is checked by calculating the ratio of the two products in Eq. (S16). We denote by $\nu\big(\{b_n\}\big) = \max\ \Re e(z)$ the largest growth rate over all roots z.

If the eigenmode associated with this growth rate is roughly the same throughout the cycle (which is reasonable, because it tends to be the mode with the largest possible wavelength in the system), then the fastest growing perturbation (under our pseudo-stationary assumption) will unfurl as

$$\exp\left(\int_0^{2\pi} \frac{d\theta}{\omega} \nu\Big(\{C\ \cos(\theta + \varphi_n)\}\Big)\right) \tag{S30}$$

over a cycle, hence an effective growth rate

$$\nu_{\text{eff}}(C) = \langle \nu(\{C\ \cos(\theta_n)\})\rangle_\theta, \tag{S31}$$

where θ_n is used as a shorthand for $\theta + \varphi_n$ and the angular brackets denote an average over $\theta \in [0, 2\pi[$. We surmise that averaging $\nu_{\text{eff}}(C)$ over random, uniformly distributed phases φ_n is tantamount to averaging it over uniformly distributed θ_n in $\mathbb{R}/2\pi\mathbb{Z}$. Numerical simulations confirm that this is a decent approximation for an arbitrary set of phases $\{\varphi_n\}$ and quite a satisfactory one upon averaging over the $\{\varphi_n\}$, as shown in Fig. S4.

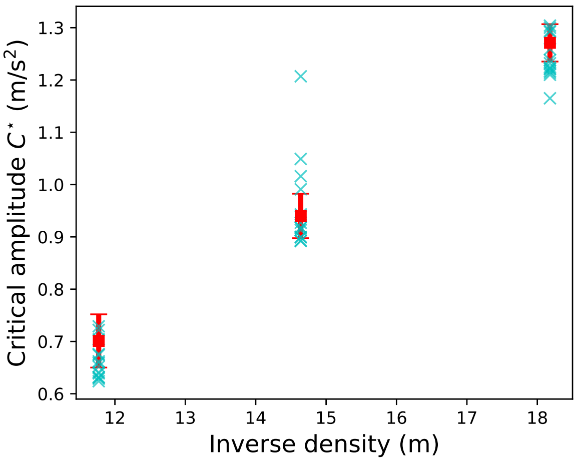

FIG. S4. Critical amplitudes $C^\star$ predicted by our extended stability analysis, using the time-dependent phases of Eq. (S30) (cyan crosses) or a random distribution of phases θ_n (red squares) at different inverse densities, for $\lambda = 0.2$ and $T = 1$ and $N = 22$ cars, with various ring perimeters. The error bars in red represent the standard errors, while the different crosses at a given density correspond to different initial phases φ_n.

D. CORRESPONDENCE BETWEEN THE CRITICAL NOISE VOLATILITY $\sigma^\star$ AND THE CRITICAL DRIVING AMPLITUDE $C^\star$

In the main text, we showed that the instability that emerges in SATG above a critical noise level $\sigma^\star$ (volatility) is mirrored by an instability in the deterministic ATG model subject to oscillatory driving $C\,\cos(\omega t + \varphi_n)$, when $C \geqslant C^\star$ ($C^\star$ is virtually insensitive to the frequency ω, provided it is low enough, typically below 0.1 rad/s); the latter instability is rationalized in Appendix C. This Appendix delves into the relation between the critical amplitudes $\sigma^\star$ and $C^\star$.

A density-independent coefficient relating $\sigma^\star$ and $C^\star$. First, we provide some numerical evidence for the existence of a density-independent coefficient $A(\lambda, T) > 0$ such that $C^\star = A(\lambda, T)\,\sigma^\star$, where $A(\lambda, T)$ may depend on model parameters λ and T, but not on the car density. Note that $\sigma^\star$ and $C^\star$ do not have the same units; $A(\lambda, T)$ has dimension $\mathrm{s}^{-1/2}$.

Figure S5 supports the existence of $A(\lambda, T)$ and its independence of density for the model parameters used in the main text. The same conclusion is reached for distinct model parameters (provided that numerical simulations of SATG and the extended stability analysis both point to a clear critical amplitude, which is not always the case when one significantly departs from the baseline values).

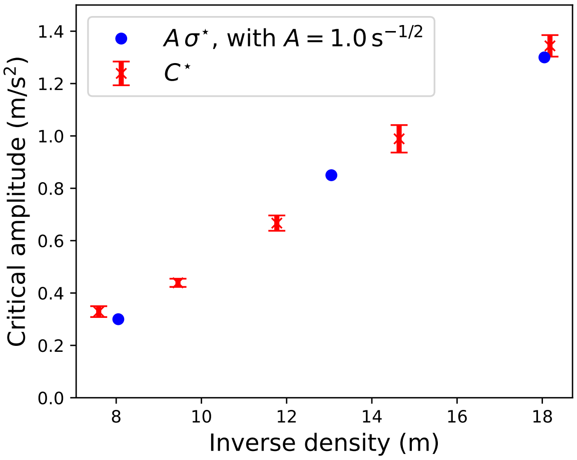

FIG. S5. Critical amplitudes $\sigma^\star$ and $C^\star$ at different inverse densities, for the SATG model parameters $\lambda = 0.2$ and $T = 1$ used in the main text. The critical volatility $\sigma^\star$ is obtained by direct numerical simulations of SATG, whereas $C^\star$ is calculated on the basis of the extended stability analysis presented in Appendix C. The coefficient $A(\lambda, T) = 1.0\,\mathrm{s}^{-1/2}$ was adjusted manually.

Analytical derivation of the coefficient $A(\lambda, T) > 0$ around the steady state. Under asynchronous (i.e., $\varphi_n \neq$ cst) oscillatory driving [4], for $C \geqslant C^\star$, the instability arises because the system is pushed to a (possibly, quasisteady) heterogeneous state which is linearly unstable (see Appendix C). The heterogeneous gaps Δx_n thus have a central role in triggering the instability. Accordingly, we strive to estimate the coefficient $A(\lambda, T)$ on a theoretical basis by equating the mean-square gap fluctuations $\langle |\Delta x|^2 \rangle$ induced by white noise, on the one hand, and by oscillatory driving, on the other hand, around the steady-state uniform flow. Below, we derive detailed expressions for these gap fluctuations, but simpler approximate formulae can be obtained by reasoning on a harmonic oscillator subjected to either thermal fluctuations or periodic driving.

We start by linearizing the equation of motion (Eq. S4), schematically written as $\dot{v}_n = F_n(\Delta x_n, v_n, v_{n+1}) + \Xi_n(t)$, around the uniform flow, in the presence of a noise term $\Xi_n(t)$ (which will be either white noise or a sinusoidal driving). We denote the deviations from the uniform flow positions and speeds by $\delta x_n(t)$ and $\delta v_n(t)$, respectively. Considering one realization of the dynamics over the time window $t \in [0, T_{sim}]$, we conduct the linear expansion in Fourier space (with $\widehat{\delta x_n}(\omega) = \int_{-\infty}^{\infty} e^{-i\omega t} \delta x_n(t) dt$ and $\widehat{\delta v_n} = i\omega \widehat{\delta x_n}$), as follows

$$-\omega^2 \widehat{\delta x_n} = F_{n,\Delta x_n} \cdot \widehat{\delta \Delta x_n} + F_{n,v_n} \cdot i\omega \widehat{\delta x_n} + F_{n,v_{n+1}} \cdot i\omega \left(\widehat{\delta x_n} + \widehat{\delta \Delta x_n} \right) + \widehat{\Xi_n}, \quad \text{(S32)}$$

where the partial derivatives read

$$F_{n,\Delta x_n} = \frac{\lambda}{T};\; F_{n,v_n} = -\lambda - \frac{1}{T};\; F_{n,v_{n+1}} = \frac{1}{T}. \tag{S33}$$

This directly leads to

$$\mathcal{N}\,\widehat{\delta x_n} = \mathcal{P}\,\widehat{\delta\Delta x_n} + \widehat{\Xi_n}, \tag{S34}$$

where $\mathcal{N}(\omega) = -\omega^2+i\omega\lambda$ and $\mathcal{P}(\omega) = T^{-1}(\lambda + i\omega)$ (we dropped the (ω) dependencies out of convenience). Subtracting the foregoing equation for n from that for $n+1$ and grouping terms, we arrive at the following equation

$$(\mathcal{N}+\mathcal{P})\,\widehat{\delta\Delta x_n} = \mathcal{P}\widehat{\delta\Delta x_{n+1}} + \left(\widehat{\Xi_{n+1}} - \widehat{\Xi_n}\right), \tag{S35}$$

whose square norm gives the mean-square gap fluctuations

$$|\mathcal{N}+\mathcal{P}|^2 \|\widehat{\delta\Delta x_n}\|^2 = |\mathcal{P}|^2\,\|\widehat{\delta\Delta x_{n+1}}\|^2 + \mathrm{CrCor} + \left(\left\|\widehat{\Xi_{n+1}}\right\|^2 + \left\|\widehat{\Xi_n}\right\|^2\right), \tag{S36}$$

where we have used the shorthand $\|\bullet\|^2 = \langle|\bullet|^2\rangle$ for the average of the square norm over realizations. The second term on the right-hand side of Eq. (S36), $\mathrm{CrCor} = 2\mathcal{R}e\left[\mathcal{P}\left\langle\widehat{\delta\Delta x_{n+1}}\left(\overline{\widehat{\Xi_{n+1}}} - \overline{\widehat{\Xi_n}}\right)\right\rangle\right]$, can be simplified by multiplying Eq. S34 by $\overline{\widehat{\Xi_n}}$, averaging over realizations, and neglecting the effect of the noise affecting one car k on the *car in front* of it [5], $k+1$, viz. $\forall k$, $\left\langle\widehat{\delta x_{k+1}}\;\overline{\widehat{\Xi_k}}\right\rangle \approx 0$, so that (writing $n = k+1$)

$$\forall k,\; -(\mathcal{N}+\mathcal{P})\left\langle\widehat{\delta\Delta x_{k+1}}\;\overline{\widehat{\Xi_{k+1}}}\right\rangle \approx \|\widehat{\Xi_{k+1}}\|^2 \tag{S37}$$

and

$$\mathrm{CrCor} \approx -2\|\widehat{\Xi_{n+1}}\|^2\mathcal{R}e\left(\frac{\mathcal{P}}{\mathcal{N}+\mathcal{P}}\right), \tag{S38}$$

where the noise on car k is assumed to have no effect on the cars $k+1$ and $k+2$ *in front of* it.

Summing Eq. (S36) over n, we deduce a relationship between $\left\|\widehat{\delta\Delta x}\right\|^2 = \frac{1}{N}\sum_n \left\|\widehat{\delta\Delta x_n}\right\|^2$ and $\|\widehat{\Xi}\|^2 = \frac{1}{N}\sum_n \|\widehat{\Xi}_n\|^2$:

$$\left\|\widehat{\delta\Delta x}\right\|^2 = 2\frac{1-\mathcal{R}e\left(\frac{\mathcal{P}}{\mathcal{N}+\mathcal{P}}\right)}{|\mathcal{N}+\mathcal{P}|^2 - |\mathcal{P}|^2}\|\widehat{\Xi}\|^2 \tag{S39}$$

$$= \overbrace{\frac{2}{\omega^4+\omega^2\left(\lambda^2+T^{-2}\right)+\lambda^2T^{-2}}}^{f(\omega,\lambda,T)}\|\widehat{\Xi}\|^2. \tag{S40}$$

We have checked that the above formula matches our expectations in the limit-cases $\lambda, T^{-1} \ll \omega$. Besides, we observe that low-frequency vibrations are equally weighted ($f \to 2T^2/\lambda^2$ as $\omega \to 0$), whereas high frequencies are damped because of the finite response time ($f \to 0$ for $\omega \gg \lambda$). In practice, for the model parameters of the main text, the crossover regime extends over barely a decade.

Now, we make use of Parseval's theorem to come back to real time space,

$$\langle(\delta\Delta x)^2(t)\rangle = (2\pi T_{sim})^{-1}\int_{-\infty}^{\infty}\|\widehat{\delta\Delta x}(\omega)\|^2 d\omega \tag{S41}$$

$$= (2\pi T_{sim})^{-1}\int_{-\infty}^{\infty} f(\omega,\lambda,T)\|\widehat{\Xi}\|^2 d\omega. \tag{S42}$$

Finally, equating the mean-square gap fluctuations $\langle(\delta\Delta x)^2(t)\rangle$ obtained with white noise of volatility σ (in which case $\|\widehat{\Xi}\|^2 = \sigma^2 T_{sim}$) with those obtained under periodic driving (in which case

$$\Xi_n(t) = C\,\cos(\tilde{\omega}t + \varphi_n),\text{ i.e., } \widehat{\Xi_n}(\omega) = \pi\,C[e^{i\varphi_n}\,\delta(\omega-\tilde{\omega}) + e^{-i\varphi_n}\,\delta(\omega+\tilde{\omega})], \tag{S43}$$

where $\delta(\bullet)$ tends to a Dirac distribution), we arrive at our final result

$$C^2 = A^2(\lambda, T)\,\sigma^2, \text{ where } A^2(\lambda, T) = \frac{1}{\pi}\int_{-\infty}^{\infty} \frac{f(\omega, \lambda, T)}{f(\tilde{\omega}, \lambda, T)} d\omega \simeq \frac{\lambda}{1 + T\lambda}. \tag{S44}$$

In the last line, we assumed the limit $\tilde{\omega}^2 \ll \min(\lambda^2, T^{-2})$ and we made use of the following identity (obtained from *Mathematica*): $\int_{-\infty}^{\infty} \frac{1}{\omega^4 + (\lambda^2 + T^{-2})\omega^2 + \lambda^2 T^{-2}} d\omega = \pi \frac{T}{\lambda\,(\lambda + T^{-1})}$.

Comparison of the theoretically derived coefficient with the actual coefficient. Let us now compare the theoretically derived coefficients $A(\lambda, T)$ (Eq. S44) with those obtained numerically as ratios between $\sigma^\star$ and the critical amplitude $C^\star$ given by our extended stability analysis, under the random phase approximation of Eq. S31. The results are presented in Table I.

The first observation is that the agreement is not strictly quantitative; the observed coefficients $A(\lambda, T)$ are typically two or three times larger than their theoretical counterparts. That being said, the theoretical estimates have the correct order of magnitude and, perhaps more importantly, they mirror the main trends observed numerically, namely:

- The coefficients do not depend on the density,
- They are virtually insensitive to the frequency $\tilde{\omega}$ of the periodic driving, up to a value around 0.1 rad/s (for $\lambda = 0.2\,\mathrm{s}^{-1}$),
- For $\lambda = 0.2\,\mathrm{s}^{-1}$, they are approximately constant, regardless of the value of T
- The coefficients get larger for $\lambda = 1\,\mathrm{s}^{-1}$ (about twice larger in the observations, somewhat less in the theoretical predictions), especially for $T = 0.5$ s.

	T=0.5	T=1	T=2
Observed values			
$\lambda = 0.2$	$\approx 1.1 a_n$	$a_n \hat{=} 1.0$	$\simeq a_n$
$\lambda = 1$	$\approx 2.7 a_n$	$\simeq 1.9 a_n$	
Theoretical values (Eq. S44)			
$\lambda = 0.2$	$1.05\, a_t$	$a_t \hat{=} 0.41$	$0.93\, a_t$
$\lambda = 1$	$2.0\, a_t$	$1.73\, a_t$	

TABLE I. Actual values of the prefactor $A(\lambda, T)$. To highlight the variations with λ, T, we present the values of $A(\lambda, T)$ relative to a_n, $a_t = A(\lambda = 0.2, T = 1)$, the values observed numerically or found theoretically for $\lambda = 0.2$ and $T = 1$.

[1] Y. Sugiyama, M. Fukui, M. Kikuchi, K. Hasebe, A. Nakayama, K. Nishinari, S.-i. Tadaki, and S. Yukawa, Traffic jams without bottlenecks—experimental evidence for the physical mechanism of the formation of a jam, New Journal of Physics. **10**, 033001 (2008).

[2] D. Ngoduy, Effect of the car-following combinations on the instability of heterogeneous traffic flow, Transportmetrica B: Transport Dynamics **3**, 44 (2015).

[3] P. Khound, P. Will, A. Tordeux, and F. Gronwald, Extending the adaptive time gap car-following model to enhance local and string stability for adaptive cruise control systems, Journal of Intelligent Transportation Systems **27**, 36 (2023).

[4] Note that an instability is also found for synchronous oscillations $\varphi_n =$ cst, but at *much* higher σ.

[5] Even though this assumption appears quite natural (because such a putative effect would be mediated by all other cars in the ring), it may generate some inaccuracies at low frequencies ω for oscillatory noise. When comparing our analytical predictions of gap standard deviations with simulations of the *linearized* system, some mismatch (of up to 50%) is indeed observed in some parameter ranges, at low frequencies ω.
